# Embryological and clinical predictors of implantation and live birth in 3000 single euploid embryo transfer cycles: a retrospective cohort study

**DOI:** 10.1007/s00404-026-08468-2

**Published:** 2026-05-21

**Authors:** Gulcin Ozkara, Hakan Kadir Yelke, Caroline Pirkevi Cetinkaya, Yesim Kumtepe Colakoglu, Semra Kahraman

**Affiliations:** 1 Department of Medical Biology, Faculty of Medicine, Bezmiâlem Vakıf Üniversitesi, Adnan Menderes Bulv. Vatan Cd. No:113, Fatih, 34093 Istanbul, Turkey; 2 Istanbul Memorial Sisli Hospital ART and Reproductive Genetics Center, Istanbul, Turkey

**Keywords:** Single euploid embryo transfer, Cryo-survival, Embryo morphology, Implantation, Live birth, IVF

## Abstract

**Purpose:**

Although single euploid embryo transfer (SEET) improves implantation and live birth (LB) rates, unsuccessful outcomes may still occur despite the transfer of morphologically high-quality euploid embryos. This study aimed to identify post-thaw embryological and clinical factors associated with implantation and LB outcomes.

**Methods:**

This retrospective cohort study included 3000 frozen–thawed SEET cycles performed at a single center. Post-thaw embryological parameters evaluated were inner cell mass (ICM) and trophectoderm (TE) grades, expansion grade, presence of necrotic areas (NA) and excluded/extruded blastomeres (EB), embryo biopsy/freezing/transfer day (Day 5 vs. Day 6), cryo-survival rate (≥90% vs. <90%), and re-expansion status. Clinical variables included maternal age, body mass index (BMI), and number of previous IVF cycles. Outcomes were analyzed with adjustment for endometrial preparation using modified natural cycle (mNC) or hormone replacement cycle (HRC) protocols. Multivariate logistic regression analysis was performed to identify independent predictors of implantation and LB.

**Results:**

Higher cryo-survival (≥90%), Day 5 embryo biopsy/freezing/transfer, and post-thaw re-expansion were associated with increased pregnancy rates (*p*<0.05). Higher embryo quality, particularly A-grade ICM, was independently associated with increased LB rates (*p*<0.05). The presence of NA and EB in thawed euploid blastocysts was significantly associated with reduced cryo-survival and lower LB outcomes (*p*<0.05). Maternal age <35 years was associated with a higher likelihood of LB compared with age ≥43 years (OR: 0.291, 95% CI 0.149–0.569, *p*<0.001). LB rates were significantly higher in mNC-prepared cycles compared with HRC (85.2% vs. 71.8%, *p*<0.001). Lower BMI and fewer previous IVF cycles were also associated with improved LB outcomes.

**Conclusion:**

Post-thaw embryological competence and maternal characteristics significantly influence implantation and live birth outcomes, even in euploid embryo transfers. Comprehensive evaluation of thawed embryo morphology combined with individualized endometrial preparation may optimize clinical outcomes following SEET.

## Introduction

Selecting embryos with the highest implantation potential is essential for achieving successful outcomes in assisted reproductive techniques (ART). Euploid embryos with high morphological quality represent one of the most significant determinants of implantation success. Currently, the two most widely adopted strategies for selecting the most competent embryo in ART are preimplantation genetic testing for aneuploidy (PGT-A) and morphological assessment, including morphokinetic evaluation and artificial intelligence (AI)-based selection [1].

Although endometrial receptivity cannot be directly measured with absolute precision, the use of standardized protocols for endometrial preparation in frozen embryo transfer (FET) cycles—particularly in single euploid transfer (SEET) settings—reduces the variability related to endometrial factors. This standardization allows embryological variables to emerge as more prominent predictors of clinical outcomes [2]. Nevertheless, unsuccessful implantation and pregnancy outcomes may still occur despite the transfer of morphologically high-quality euploid embryos [3–5]. Therefore, identifying risk factors that influence clinical outcomes following embryo thawing in euploid embryo transfer cycles remains of significant clinical relevance.

The selection of the most competent embryo to achieve successful ART outcomes has been extensively studied, and several predictive models incorporating morphological and/or morphokinetic parameters have been proposed [6–8]. Among embryos undergoing PGT-A, the efficiency of embryo cryopreservation and thawing is a critical determinant of implantation success. With the transition from slow-freezing methods to vitrification, cryo-survival rates have markedly improved, resulting in increased clinical application of frozen–thawed euploid embryo transfer [9].

Previous studies have examined factors influencing post-thaw embryo viability and clinical outcomes, including the timing of vitrification after trophectoderm biopsy [10, 11], post-thaw re-expansion dynamics [12–15], the day of embryo transfer [5, 16–20], embryo morphological quality [1, 8, 11, 19–23], the presence of excluded or extruded blastomeres (EB) [24, 25], cryo-survival rate [11, 21], maternal age [5, 8, 16–18, 22, 26, 27], and body mass index (BMI) [5, 8, 23, 28–31]. However, substantial heterogeneity in study populations and clinical protocols limits the generalizability of these findings.

Therefore, the present study aimed to retrospectively evaluate the impact of post-thaw embryological parameters on live birth (LB) outcomes following single euploid embryo transfer (SEET) in a large cohort, while minimizing confounding factors related to endometrial receptivity by excluding patients with recurrent pregnancy loss (RPL) and recurrent implantation failure (RIF). In addition, subgroup analyses were performed according to endometrial preparation (EP) protocols to further control for potential endometrial influences on ART outcomes.

## Materials and methods

### Study design

**Fig. 1:**
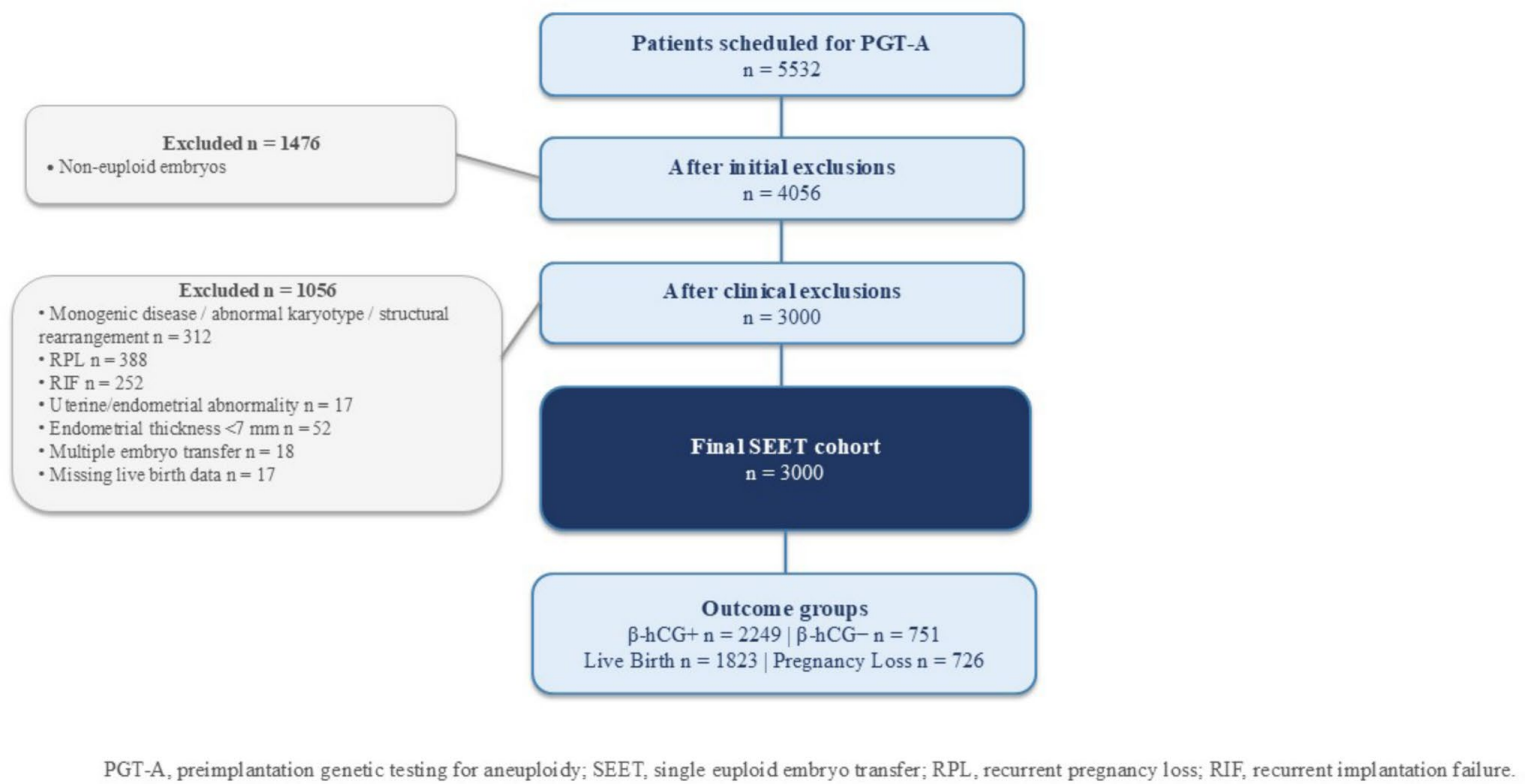
Flow diagram of patient and cycle selection in the study

This retrospective cohort study was conducted at the Istanbul Memorial Sisli Hospital ART and Reproductive Genetics Center. Data were obtained from the electronic medical records system and included 3000 PGT-A cycles with complete data and known birth outcomes performed between January 2017 and October 2021. The main indications for PGT-A were advanced maternal age (36.6%), aneuploidy screening to reduce time to pregnancy (32.3%), severe male factor infertility (20.1%), and a history of fetal anomaly (11%). Although the dataset was derived from the same ART program as previous studies, the present cohort was specifically defined based on SEET cycles with detailed post-thaw embryological assessment and was analyzed with different study objectives and variables. A flowchart of SEET cycle selection is shown in Fig. 1. The study protocol was approved by the Institutional Review Board of Istanbul Memorial Sisli Hospital (decree no. 03.06.2023/003).

Patients were stratified according to ART outcomes as β-hCG-positive versus β-hCG-negative and live birth (LB) versus pregnancy loss (PL). A predefined subgroup analysis was also performed based on endometrial preparation (EP) protocols, comparing modified natural cycles (mNC) with hormone replacement cycles (HRC).

**Fig. 2:**
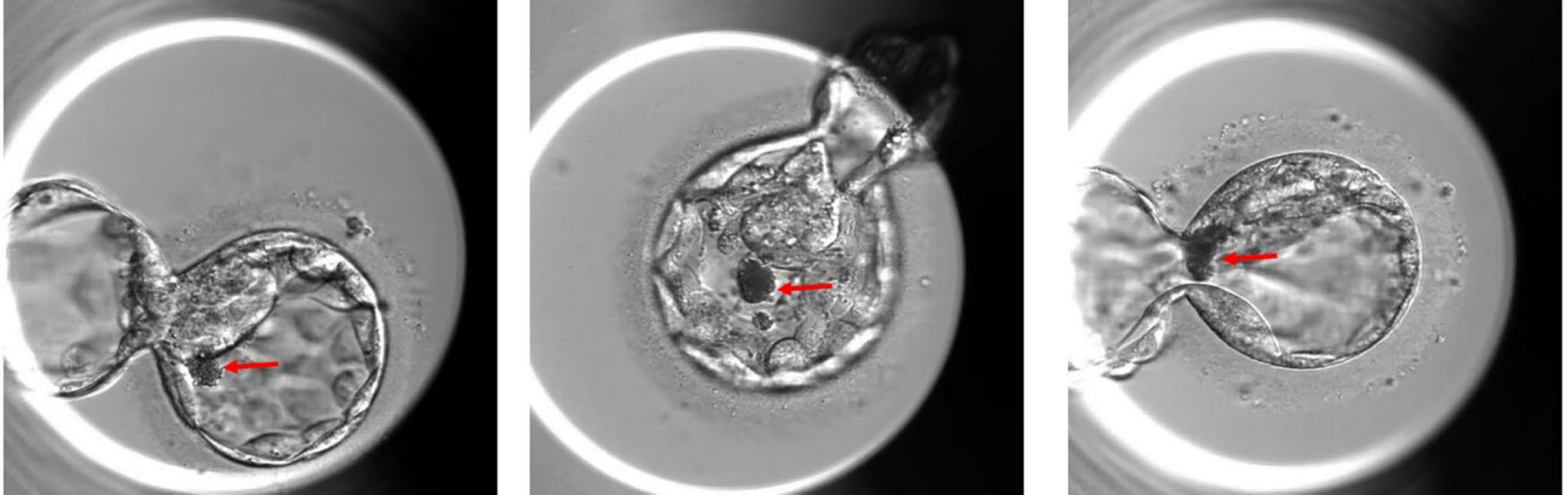
Examples of blastocysts with necrotic areas

**Fig. 3:**
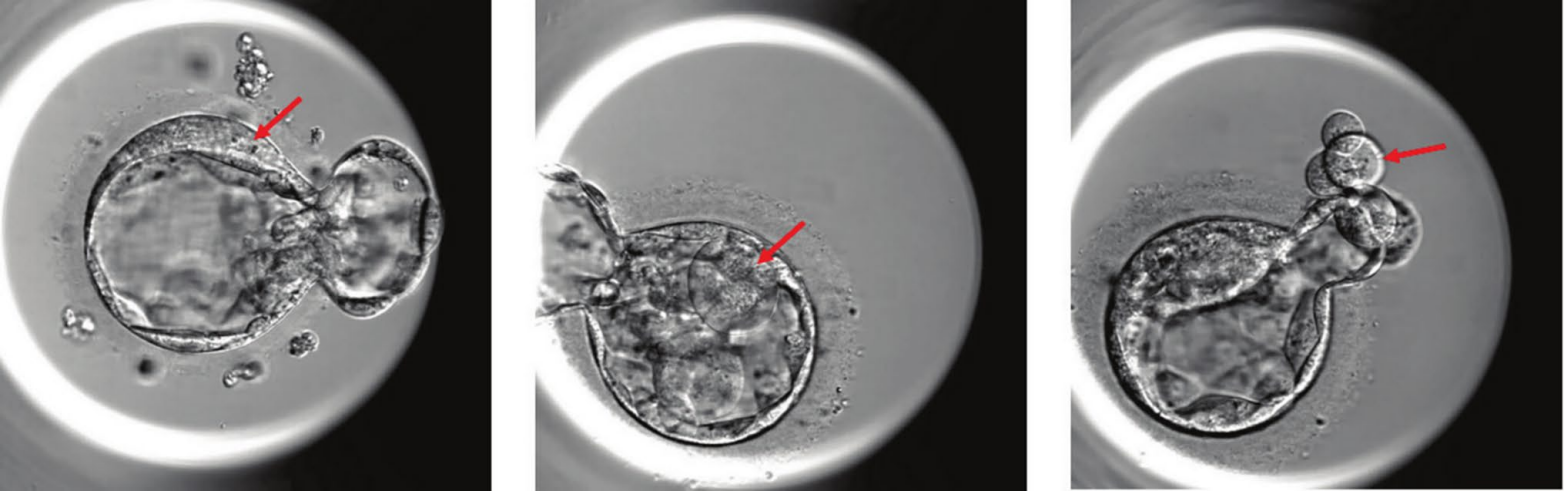
Blastocysts with excluded/extruded blastomere(s)

Post-thaw embryological parameters were systematically evaluated, including cryo-survival rate (≥90% vs. <90%), blastocyst re-expansion dynamics, embryo morphological quality (top-quality [TQ], good-quality [GQ], and moderate/poor-quality [MQ/PQ]), inner cell mass (ICM) and trophectoderm (TE) grades (A, B, or C), presence of necrotic areas (NA; Fig. 2), excluded or extruded blastomeres (EB; Fig. 3), and the day of trophectoderm biopsy and cryopreservation (Day 5 or Day 6). Blastocyst re-expansion was assessed after warming at 0, 2, and 4 h. The 0 h assessment reflected the immediate post-warming expansion status, which corresponded to the re-expansion observed prior to vitrification following a 1-h incubation after trophectoderm biopsy. All embryos were vitrified following a standardized 1 h post-biopsy incubation period.

The inclusion criteria were endometrial thickness ≥7 mm, SEET, and absence of RPL, RIF, or congenital/acquired uterine or endometrial abnormalities. Exclusion criteria included use of PGT-A methods other than next-generation sequencing (NGS), embryos cryopreserved outside our center, untreated endocrinological disorders, monogenic diseases, abnormal karyotypes or structural chromosomal rearrangements, and endometrial thickness <7 mm at the time of embryo transfer.

### Controlled ovarian stimulation protocol

All patients underwent a GnRH antagonist protocol with gonadotropin doses individualized according to patient characteristics. Ovarian stimulation was performed using recombinant follicle-stimulating hormone (rFSH; Gonal-f ®, Merck, Switzerland), a combination of rFSH and recombinant luteinizing hormone (rLH; Luveris ®, Merck, Switzerland), or human menopausal gonadotropin (hMG; Ferring, Switzerland). GnRH antagonists (Cetrotide ®, Merck-Serono, Switzerland; or Orgalutran ®, MSD, The Netherlands) were initiated when at least one follicle reached a diameter of ≥14 mm. Follicular development was monitored at regular intervals using transvaginal ultrasonography. Final oocyte maturation was triggered with 250 μg recombinant human chorionic gonadotropin (rhCG; Ovitrelle ®, Merck-Serono, Switzerland) when at least two dominant follicles measured ≥17 mm. Oocyte retrieval (OPU) was performed 36 h later under transvaginal ultrasound guidance.

### Embryo culture and classification of embryos

Embryos were cultured in a single-step embryo culture medium (Life Global ®, Belgium) supplemented with 5% human serum albumin (HSA; Life Global®, Belgium) using a time-lapse monitoring system (EmbryoScope ®; Vitrolife, Sweden) or a benchtop incubator under 6% CO₂, 5% O₂, and 37 °C, with a pH range of 7.26–7.30, for 5–6 days. Culture dishes (EmbryoSlide ®; Vitrolife, Sweden) were covered with 25 μl medium per well and a total of 1.5 ml paraffin oil (Life Global ®, Belgium).

Embryo morphology was evaluated before vitrification and after warming according to the Gardner classification system. Embryos were categorized as TQ (4AA/5AA/6AA), GQ (3AA/4BA/4AB/4BB/5AB/5BA/5BB/6AB/6BA/6BB), and MQ/PQ (all remaining embryos).

### Trophectoderm biopsy procedure

Artificial hatching of the zona pellucida was performed on Day 3 of embryo culture using a diode laser (RI Saturn 3, England). Trophectoderm biopsy was conducted by aspirating five to eight trophectoderm cells using a biopsy pipette with an inner diameter of 30 μm (Origio, Denmark) via the flicking method.

### Embryo cryopreservation and warming

One hour after trophectoderm biopsy, the blastocysts were vitrified using Kitazato vitrification media (Kitazato, Japan) with a Cryotop® carrier, according to the manufacturer’s instructions. Following PGT-A, the euploid blastocysts were warmed with Kitazato warming media (Kitazato, Japan).

### Genetic analysis

PGT-A was performed using NGS with the ReproSeq kit (Thermo Fisher Scientific, USA) on the Ion Torrent™ S5™ platform (Thermo Fisher Scientific, USA). Data analysis was conducted using the Ion Reporter software versions 5.2 and 5.6 (Thermo Fisher Scientific, USA).

### Endometrial preparation and embryo transfers

Endometrial preparation protocols (mNC or HRC) were selected based on individual cycle characteristics and clinician preference, including menstrual regularity, ovulatory status, and prior cycle history, as previously described by Ozer et al. [7]. Embryo transfer was scheduled according to the duration of progesterone exposure to ensure synchronization between endometrial receptivity and embryo developmental stage. Blastocysts biopsied and vitrified on Day 5 or Day 6 were transferred after 5 or 6 days of progesterone administration, respectively. Luteal phase support with vaginal progesterone was continued until the 10th gestational week.

A serum β-hCG concentration >20 IU/L measured 9 days after embryo transfer was considered indicative of biochemical pregnancy. Clinical pregnancy was defined as the visualization of an intrauterine gestational sac by transvaginal ultrasonography between the 6th and 8th gestational weeks.

Biochemical pregnancy loss (BPL) was defined as a decline in β-hCG levels following a positive pregnancy test, whereas clinical pregnancy loss (CPL) referred to pregnancy termination after ultrasound confirmation of a gestational sac. Although BPL and CPL represent biologically distinct events with different underlying mechanisms, for statistical purposes, both entities were categorized as pregnancy loss (PL). Live birth (LB) was defined as the delivery of a live infant beyond 24 weeks of gestation.

### Statistical analysis

Statistical analyses were performed using IBM SPSS software (version 25). Data distribution was assessed using the Kolmogorov–Smirnov test. Continuous variables were summarized as mean ± standard deviation and compared using the Mann–Whitney U test, whereas categorical variables were expressed as numbers (n) and percentages (%) and compared using the Chi-square test.

To identify independent predictors of implantation (β-hCG positivity) and live birth, multivariable logistic regression analyses were performed using a stepwise backward selection approach. Variables that were found to be statistically significant in univariable analyses (*p*<0.05) were included in the initial models. These variables included maternal age, BMI, number of previous IVF cycles, endometrial preparation protocol, cryo-survival rate, re-expansion status, embryo quality, ICM and TE grades, presence of necrotic areas and excluded/extruded blastomeres, and Day 5 versus Day 6 biopsy/cryopreservation/transfer. Adjusted odds ratios (ORs) with 95% confidence intervals (CIs) were calculated. Model fit was assessed using the Hosmer–Lemeshow goodness-of-fit test. The cryo-survival threshold of ≥90% was defined according to the ESHRE Vienna consensus [32]. This approach enabled adjustment for potential confounding effects among the included variables.

## Results

Baseline demographic and clinical characteristics of the study population are summarized in Table 1. Maternal age at the time of transfer (years) was significantly lower in the βhCG-positive group compared with the βhCG-negative group (33.71±4.87 vs. 34.21±4.75, *p*=0.009). Although BMI (kg/m^2^) did not differ between βhCG-positive and -negative groups, it was significantly lower in patients who achieved live birth (LB) compared with those experiencing pregnancy loss (PL) (24.19±4.16 vs. 24.68±4.28, *p*=0.029).

**Table 1:** Baseline demographic and cycle characteristics of patients undergoing single euploid embryo transfer (SEET)

	βhCG (+) (*n*=2249)	βhCG (-) (*n*=751)	*p*-Value (βhCG + vs. -)	Live birth (*n*=1823)	Pregnancy loss (*n*=426)	*p*-Value (LB vs. PL)
Maternal age at SEET (years)	**33.71 ± 4.87**	34.21 ± 4.75	**0.009**	33.68 ± 4.78	33.82 ± 5.23	0.640
BMI (kg/m2)	24.28 ± 4.18	24.49 ± 4.15	0.214	**24.19 ± 4.16**	24.68 ± 4.28	**0.029**
Infertility duration (years)	**4.39 ± 3.59**	4.92 ± 3.81	**<0.001**	4.33 ± 3.54	4.63 ± 3.78	0.243
Previous IVF cycles (n)	**3.44 ± 2.47**	4.59 ± 2.84	**<0.001**	**3.22 ± 2.35**	4.4 ± 2.76	**<0.001**
AMH (ng/ml)	**2.98 ± 2.41**	2.73 ± 2.22	**0.013**	2.95 ± 2.35	3.1 ± 2.66	0.289
Basal FSH (mIU/mL)	8.11 ± 2.87	7.94 ± 2.68	0.654	8.11 ± 2.92	8.09 ± 2.68	0.947
Basal E2 (ng/L)	41.14 ± 22.20	41.78 ± 23.29	0.791	40.94 ± 21.25	41.97 ± 25.9	0.930
Basal LH (IU/L)	6.40 ± 2.65	6.31 ± 2.97	0.181	6.36 ± 2.63	6.54 ± 2.76	0.381
E2 on trigger day (ng/L)	**2844.36 ± 1909.49**	2630.79 ± 1711.25	**0.044**	2834.39 ± 1920.43	2887.10 ± 1863.59	0.392
LH on trigger day (IU/L)	2.43 ± 1.94	2.47 ± 1.93	0.550	2.43 ± 1.97	2.44 ± 1.78	0.399
Total gonadotropin dose (IU)	2239.23 ± 830.24	2290.26 ± 865.14	0.119	2232.88 ± 829.88	2266.45 ± 832.2	0.347
Daily gonadotropin dose (IU)	247.83 ± 70.76	248.03 ± 65.67	0.591	247.77 ± 70.84	248.09 ± 70.53	0.654
Duration of COS (Days)	9.01 ± 1.66	9.17 ± 1.70	0.051	8.99 ± 1.65	9.12 ± 1.68	0.163
Total follicle count (*n*)	**12.12 ± 6.41**	11.05 ± 5.84	**<0.001**	12.13 ± 6.42	12.07 ± 6.34	0.965
≥17 mm follicle count (*n*)	**7.25 ± 4.50**	6.60 ± 4.17	**0.001**	7.25 ± 4.56	7.25 ± 4.26	0.628
Total oocytes retrieved (*n*)	**13.28 ± 8.34**	12.15 ± 7.64	**0.001**	13.28 ± 8.37	13.31 ± 8.21	0.763
Mature (MII) oocytes (*n*)	**11.62 ± 7.21**	10.71 ± 6.52	**0.004**	11.62 ± 7.19	11.62 ± 7.28	0.990
Fertilized oocyte (2PN) (*n*)	**9.59 ± 6.17**	8.81 ± 5.57	**0.004**	9.6 ± 6.15	9.55 ± 6.25	0.811
EP method						
mNC	1557 (75.8%)	496 (24.2%)	0.104	**1326 (85.2%)**	231 (14.8%)	**<0.001**
HRC	692 (73.1%)	255 (26.9%)		**497 (71.8%)**	195 (28.2%)	

*AMH* anti-Müllerian hormone, *COS* controlled ovarian stimulation, *E2* estradiol, *EP* endometrial preparation, *FET* frozen embryo transfer, *FSH* follicle-stimulating hormone, *HRC* hormone replacement cycle, *LH* luteinizing hormone, *MII* metaphase II, *mNC* modified natural cycle, *PN* pronucleus, *SEET* single euploid embryo transfer

All cycles included single euploid embryo transfers confirmed by next-generation sequencing (NGS). Only patients with endometrial thickness ≥7 mm and without recurrent pregnancy loss (RPL) or recurrent implantation failure (RIF) were included

Continuous variables were compared using the Mann–Whitney *U* test, and categorical variables were compared using the Chi-square test. Data are presented as mean ± standard deviation (SD) or number (*n*) and percentage (%). Statistically significant results (*p*<0.05) are shown in bold.

The duration of infertility was similar between the LB and PL groups; however, it was lower in the βhCG-positive group than in the βhCG-negative group (4.39±3.59 years vs. 4.92±3.8, *p*<0.001). The number of previous IVF cycles was also observed to be lower in the βhCG-positive and LB groups compared to the βhCG-negative and PL groups (3.44±2.47 vs. 4.59±2.84 and 3.22±2.35 vs. 4.40±2.76, *p*<0.001, respectively). Ovarian reserve and stimulation parameters including anti-Müllerian hormone (AMH) levels, estradiol (E2) levels on the trigger day, follicle counts, and oocyte yield were significantly higher (*p*<0.05) in the βhCG-positive group, whereas no significant differences (*p*>0.05) were observed between the LB and PL groups for these parameters. Total gonadotropin dose, daily dose, and duration of controlled ovarian stimulation did not differ significantly between outcome groups (*p*>0.05) (Table 1).

Regarding endometrial preparation, most frozen embryo transfers were performed using mNC (69.2%). While pregnancy rates were comparable between mNC and HRC (75.8% vs 73.1%; *p*>0.05), the LB rate was significantly higher in patients prepared with mNC compared with HRC (85.2% vs. 71.8%; *p*<0.001) (Table 1).

Post-thaw embryological characteristics of euploid embryos are presented in Table 2. A cryo-survival rate ≥90% was significantly more frequent in both the β-hCG-positive and LB groups compared with the β-hCG-negative and PL groups, respectively. (99.6% vs. 97.1%, *p*<0.001 and 99.8% vs. 99.1%, *p*=0.025, respectively). The absence of NA and EB was also significantly more common among embryos in βhCG-positive and LB groups (*p*<0.001) (Table 2).

**Table 2:** Post-thaw embryological characteristics of euploid embryos according to β-hCG positivity and live birth outcomes

	βhCG (+) (*n*=2249)	βhCG (-) (*n*=751)	*p*-Value (βhCG + vs. -)	Live Birth (*n*=1823)	Pregnancy loss (*n*=426)	*p*-Value (LB vs. PL)
Cryo-survival rate (≥90%) (*n*) (%)	**2240 (99.6%)**	729 (97.1%)	**<0.001**	**1819 (99.8%)**	422 (99.1%)	**0.025**
Absence of NA (*n*) (%)	**2045 (90.9%)**	639 (85.1%)	**<0.001**	**1682 (92.3%)**	363 (85.2%)	**<0.001**
Absence of EB (*n*) (%)	**1926 (85.6%)**	599 (79.8%)	**<0.001**	**1587 (87.1%)**	339 (79.6%)	**<0.001**
Presence of re-expansion after thawing (*n*) (%)						
0 h	**2028 (90.2%)**	633 (84.3%)	**<0.001**	**1657 (90.9%)**	371 (87.1%)	**0.018**
2 h	**2154 (95.8%)**	684 (91.1%)	**<0.001**	1748 (95.9%)	406 (95.3%)	0.592
4 h	2042 (90.8%)	668 (88.9%)	0.138	1645 (90.2%)	397 (93.2%)	0.057
TB/freezing/transfer day (*n*) (%)						
Day 5	**2121 (94.3%)**	661 (88%)	**<0.001**	1724 (94.6%)	397 (93.2%)	0.269
Day 6	**128 (5.7%)**	90 (12%)		99 (5.4%)	29 (6.8%)	
Embryo quality (*n*) (%)						
TQ	**1410 (62.7%)**	360 (47.9%)		**1171 (64.2%)**	238 (54.9%)	
GQ	**774 (34.4%)**	325 (43.3%)	**<0.001**	**608 (33.4%)**	166 (39%)	**<0.001**
MQ+PQ	**65 (2.9%)**	66 (8.8%)		**44 (2.4%)**	22 (5.2%)	
ICM grade						
A	**2071 (92.1%)**	632 (84.2%)	**<0.001**	**1695 (93%)**	376 (88.2%)	**<0.001**
B/C	**178 (7.9%)**	119 (15.8%)		**128 (7%)**	50 (11.8%)	
TE grade						
A	**1516 (67.4%)**	420 (55.9%)	**<0.001**	**1256 (68.9%)**	260 (61.1%)	**0.002**
B/C	**733 (32.6%)**	331 (44.1%)		**567 (31.1%)**	166 (38.9%)	
Expansion grade						
6	**1152 (51.2%)**	354 (47.2%)		773 (42.4%)	206 (48.4%)	
5	**443 (19.7%)**	140 (18.6%)	**<0.001**	432 (23.7%)	82 (19.2%)	0.168
4	**575 (25.6%)**	191 (25.5%)		551 (30.2%)	117 (27.5%)	
≤3	**79 (3.5%)**	66 (8.7%)		67 (3.7%)	21 (4.9%)	
Freezing time post-ICSI (h)	**116.36 ± 3.29**	117.11 ± 4.93	**0.002**	116.34 ± 3.30	116.48 ± 3.25	0.470

Post-thaw assessment included cryo-survival rate, re-expansion status at 0, 2, and 4 h after warming, embryo quality categories, ICM and TE grades, and the presence of NA and EB. Mann–Whitney *U* test and Chi-square test were used to compare continuous variables and categorical variables, respectively. Data are presented as mean ± standard deviation or *n* (%). Statistically significant values (*p*<0.05) are shown in bold

*EB* excluded/extruded blastomere(s), *GQ* good quality, *ICM* inner cell mass, *h* hours, *MQ* moderate quality, *n* number, *NA* necrotic area, *PQ* poor quality, *TB* trophectoderm biopsy, *TE* trophectoderm, *TQ* top quality

The proportion of TQ embryos was significantly more prevalent in both the βhCG-positive and the LB groups (62.7% vs. 47.9% and 64.2% vs. 54.9%, *p*<0.001), whereas MQ/PQ embryos were lower (2.9% vs. 8.8% and 2.4% vs. 5.2%, p<0.001). Similarly, embryos with grade A ICM and TE were significantly more frequent in the βhCG-positive and the LB groups (ICM grade A: 92.1% vs. 84.2%, *p*<0.001 and 93% vs. 88.2%, *p*=0.001; TE grade A: 67.4% vs. 55.9%, *p*<0.001 and 68.9% vs. 61.1%, *p*=0.002, respectively). Although the expansion grade of euploid blastocysts was higher in the βhCG-positive cycles (*p*<0.001), this difference did not reach statistical significance for LB outcomes (*p*>0.05) (Table 2).

Day 5 trophectoderm biopsy, cryopreservation, and embryo transfer were more frequently observed in the β-hCG-positive group (94.3% vs. 88%, *p*<0.001), whereas no significant difference was found between the LB and PL groups (94.6% vs. 93.2%, *p*>0.05). The duration from ICSI to blastocyst vitrification was comparable between the LB and PL groups (116.34h±3.30 vs. 116.48h±3.25, *p*>0.05) but was slightly shorter in β-hCG-positive cycles (116.36±3.29 vs 117.11±4.93, *p*=0.002) (Table 2).

To determine the key factors affecting implantation and LB, a stepwise backward logistic regression analysis was performed using categorical parameters that were found significant (*p*<0.05) by the Chi-square test. The dependent variables were βhCG positives (vs. negatives) and LB (vs. PL), and independent variables were selected from the statistically significant parameters in each comparison. Accordingly, cryo-survival ≥90% (*p*=0.003, OR: 0.266, 95% CI 0.112–0.634), embryo biopsy/freezing/transfer on Day 5 (*p*<0.001, OR: 0.577, 95% CI 0.427–0.780), grade A ICM compared to grade B/C (*p*=0.011, OR: 0.692, 95% CI 0.522–0.912), TQ embryos compared to GQ (*p*<0.001, OR: 0.709, 95% CI 0.586–0.858), TQ embryos compared to MQ/PQ (*p*<0.001, OR: 0.380, 95% CI 0.254–0.570) were found to be associated with positive pregnancy (Table 3). On the other hand, maternal age <35 compared with ≥43 (*p*<0.001, OR: 0.291, 95% CI 0.149–0.569), mNC compared with HRC (*p*<0.001, OR: 0.432, 95% CI 0.346-0.539), absence of NA (*p*<0.001, OR: 0.550, 95% CI 0.395–0.764), and absence of EB (*p*<0.001, OR: 0.545, 95% CI 0.410–0.723) were identified as independent predictors of LB in SEET (Table 4).

**Table 3:** Stepwise backward logistic regression model identifying predictors of positive pregnancy outcome following single euploid embryo transfer

Step 9		*p*-Value	OR	95% CI for OR	
				Lower	Upper
Post-thaw embryological parameters	Cryo-survival (<90%) (Ref. ≥90%)	**0.003**	**0.266**	**0.112**	**0.634**
	Absence of re-expansion after 2 h (Ref. presence)	0.058	0.700	0.484	1.013
	TB/Freezing/ET Day 6 (Ref. Day 5)	**<0.001**	**0.577**	**0.427**	**0.780**
	ICM Grade B/C (Ref. A)	**0.011**	**0.692**	**0.522**	**0.917**
	GQ (Ref. TQ)	**<0.001**	**0.709**	**0.586**	**0.858**
	MQ+PQ (Ref. TQ)	**<0.001**	**0.380**	**0.254**	**0.570**

Statistically significant results (*p*<0.05) are shown in bold

Model calibration was assessed using the Hosmer–Lemeshow goodness-of-fit test (*p*=0.877). The overall model was statistically significant (*p*<0.001)

*CI* confidence interval, *ET* embryo transfer, *GQ* good quality, *ICM* inner cell mass, *OR* odds ratio, *Ref*. Reference, *MQ* moderate quality, *PQ* poor quality, *TB* trophectoderm biopsy, *TQ* top quality

**Table 4:** Stepwise backward logistic regression model identifying factors associated with live birth following single euploid embryo transfer

Step 3		*p*-Value	OR	95% CI for OR	
				Lower	Upper
Maternal age	35-37 (Ref. <35)	0.346	1.150	0.860	1.537
	38-39 (Ref. <35)	0.534	1.111	0.798	1.547
	40-42 (Ref. <35)	0.863	1.032	0.718	1.485
	≥43 (Ref. <35	**<0.001**	**0.291**	**0.149**	**0.569**
Post-thaw embryological parameters	Endometrial preparation HRC (Ref. mNC)	**<0.001**	**0.432**	**0.346**	**0.539**
	Presence of necrotic area (Ref. Absence)	**<0.001**	**0.550**	**0.395**	**0.764**
	Presence of EB (Ref. Absence)	**<0.001**	**0.545**	**0.410**	**0.723**

Statistically significant results are shown in bold (*p*<0.05)

Hosmer–Lemeshow goodness-of-fit test: *p*=0.200; overall model significance: *p*<0.001

*CI* confidence interval, *EB* excluded/extruded blastomere(s), *HRC* hormone replacement cycle, *mNC* modified natural cycle, *OR* odds ratio, *Ref*. reference

Given that most transfers were performed using mNC (*n*=1557, 69.2%), subgroup analyses were conducted in this population. In mNC cycles, cryo-survival ≥90% (*p*=0.005, OR: 0.220, 95% CI 0.076–0.638), re-expansion at 2 h post-thaw (*p*=0.002, OR: 0.449, 95% CI 0.273–0.737), embryo biopsy/freezing/transfer on Day 5 (*p*=0.005, OR: 0.596, 95% CI 0.415–0.856), grade A ICM compared to grade B/C (*p*=0.006, OR: 0.616, 95% CI 0.436–0.869), TQ embryos compared to GQ (*p*=0.007, OR: 0.724, 95% CI 0.571–0.917), TQ embryos compared to MQ/PQ (*p*<0.001, OR: 0.387, 95% CI 0.236–0.635) were found to be independently associated with positive pregnancy (Table 5). The absence of NA (*p*=0.007, OR: 0.541, 95% CI 0.346–0.844), the absence of EB (*p*=0.001, OR: 0.551, 95% CI 0.387–0.783), and grade A ICM compared with grade B/C (*p*=0.040, OR: 0.613, 95% CI 0.384–0.979) remained significant predictors of LB (Table 6).

**Table 5:** Stepwise backward logistic regression analysis identifying predictors of implantation in modified natural cycle transfers

Step 8		*p*-Value	OR	95% CI for OR	
				Lower	Upper
Post-thaw embryological parameters	Cryo-survival <90% (Ref. ≥90%)	**0.005**	**0.220**	**0.076**	**0.638**
	Absence of re-expansion after 2 h (Ref. presence)	**0.002**	**0.449**	**0.273**	**0.737**
	TB/Freezing/ET Day 6 (Ref. Day 5)	**0.005**	**0.596**	**0.415**	**0.856**
	ICM B/C (Ref. A)	**0.006**	**0.616**	**0.436**	**0.869**
	GQ (Ref. TQ)	**0.007**	**0.724**	**0.571**	**0.917**
	MQ+PQ (Ref. TQ)	**<0.001**	**0.387**	**0.236**	**0.635**

Statistically significant results are shown in bold (*p*<0.05)

Hosmer–Lemeshow goodness-of-fit test: *p*=0.916; overall model significance: *p*<0.001

*CI* confidence interval, *ET* embryo transfer, *GQ* good quality, *ICM* inner cell mass, *OR* odds ratio, *Ref*. Reference, *MQ* moderate quality, *PQ* poor quality, *TB* trophectoderm biopsy, *TQ* top quality

**Table 6:** Stepwise backward logistic regression model of live birth predictors in modified natural cycle single euploid embryo transfers

Step 4		*p*-Value	OR	95% CI for OR	
				Lower	Upper
Maternal age	35-37 (Ref age<35	0.183	1.292	0.886	1.884
	38-39 (Ref age<35)	0.132	1.446	0.895	2.336
	40-42 (Ref age<35)	0.544	1.162	0.716	1.886
	≥43 (Ref age<35)	**<0.001**	**0.206**	**0.089**	**0.479**
Post-thaw embryological parameters	Presence of necrotic area (Ref. Absence)	**0.007**	**0.541**	**0.346**	**0.844**
	Presence of EB (Ref. Absence)	**0.001**	**0.551**	**0.387**	**0.783**
	ICM grade B/C (Ref. A)	**0.040**	**0.613**	**0.384**	**0.979**

Statistically significant results (*p*<0.05) are shown in bold.

Hosmer–Lemeshow goodness-of-fit test: *p*=0.309; model *p*<0.001

*CI* confidence interval, *EB* excluded/extruded blastomere(s), *ICM* inner cell mass, *OR* odds ratio, *Ref*. Reference

Finally, embryological risk factors associated with reduced cryo-survival rate (<90%) were evaluated. The presence of NA and EB, along with lower embryo quality, poorer ICM and TE grades (B/C vs A), and lower expansion grades were significantly associated with decreased post-thaw survival in euploid embryos (*p*<0.001) (Table 7).

**Table 7:** Embryological predictors of reduced cryo-survival rate

	Cryo-survival rate <90%	Cryo-survival rate ≥90%	*p*-Value
Presence of NA	**80.6%**	**9.9%**	**<0.001**
Presence EB	**58.1%**	**15.6%**	**<0.001**
ICM grade			
A	**51.7%**	**90.4%**	**<0.001**
B/C	**48.3%**	**9.6%**	
TE grade			
A	**24.1%**	**64.8%**	**<0.001**
B/C	**75.9%**	**35.2%**	
Expansion grade			
6	**29.0%**	**50.3%**	
5	**6.5%**	**19.4%**	**<0.001**
4	**16.1%**	**26.0%**	
≤3	**48.4%**	**4.3%**	
Embryo quality			
TQ	**12.9%**	**59.3%**	
GQ	**38.7%**	**36.7%**	**<0.001**
MQ+PQ	**48.4%**	**4.0%**	

Values are presented as %. Comparisons were performed using Fisher’s exact test for 2×2 variables and Pearson’s Chi-square test for variables with more than two categories

Statistically significant results (*p*<0.05) are shown in bold

*EB* excluded/extruded blastomere(s), *ICM* inner cell mass, *MQ* moderate quality, *NA* necrotic areas, *PQ* poor quality, *TQ* top quality

## Discussion

Understanding the determinants of success following single euploid embryo transfer remains a critical challenge in ART. Although chromosomal normality eliminates one of the major causes of implantation failure, unsuccessful outcomes still occur, underscoring the contribution of post-thaw embryological integrity, maternal characteristics, and endometrial preparation. The present study provides a comprehensive evaluation of these factors in a large, single-center cohort, focusing specifically on post-thaw embryological parameters and their association with implantation and live birth outcomes.

The impact of maternal age on outcomes following euploid embryo transfer remains controversial [5, 8, 16–18, 22, 23, 26, 27, 29, 33]. Several studies have reported an inverse association between increasing maternal age and implantation, ongoing pregnancy, or LB rates [16, 17, 22, 23, 26, 33]. Large-scale analyses and meta-analyses have demonstrated that maternal age ≥38 years is associated with reduced LB rates, even when euploid embryos are transferred [22, 23, 26]. In contrast, other studies have found no significant association between maternal age and pregnancy outcomes in SEET cycles [5, 18, 27, 30].

In the present study, although maternal age was lower in βhCG-positive cases, logistic regression analysis did not demonstrate a significant association between age subgroups (<35, 35–37, 38–39, 40–42, ≥43 years) and implantation. However, advanced maternal age (≥43 years) was more prevalent in the PL group than in the LB group, and maternal age <35 years was strongly associated with LB compared with ≥43 years. This association persisted in subgroup analyses restricted to mNC preparation, suggesting that extreme maternal age may adversely affect LB outcomes even in euploid embryo transfers. Consistently, previous work from our group demonstrated an increased risk of first-trimester pregnancy loss in women aged ≥35 years undergoing frozen–thawed good-quality embryo transfers [8]. The observed discrepancies among studies may be explained by differences in age cut-offs, cohort characteristics, and EP protocols. Age-related alterations in endometrial receptivity, including changes in gene expression, hormone receptor signaling, and epithelial proliferation, may contribute to impaired implantation and pregnancy maintenance despite chromosomal normality of the embryo [34, 35].

Maternal BMI also emerged as an important factor influencing outcomes [8, 29]. Elevated BMI has been associated with increased miscarriage risk and reduced ongoing pregnancy or LB rates in SEET cycles [5, 23, 29, 30], although conflicting findings exist [31]. Molecular studies suggest that obesity-related alterations in endometrial gene expression may impair implantation and early placentation [36]. These findings underscore the need for further mechanistic studies evaluating the molecular pathways linking BMI to ART outcomes.

Regarding EP methods, mNC preparation was associated with significantly higher LB rates compared with hormone replacement cycles (HRC), a finding consistent with previous reports from our group [8, 29]. Although the literature remains heterogeneous, several studies suggest that EP method per se may not directly influence LB, but rather modulates miscarriage risk or interacts with endometrial thickness and hormonal milieu [5, 27, 30, 33]. Lower estrogen exposure in mNC cycles may promote a more physiologic endometrial environment, potentially favoring implantation and placental development [37, 38]. As endometrial preparation protocols were not randomly assigned, residual confounding cannot be entirely excluded. Subgroup analyses restricted to mNC cycles were, therefore, performed to minimize protocol-related variability and to evaluate the independent contribution of embryological parameters under more homogeneous clinical conditions. However, formal interaction testing was not conducted; therefore, potential differences in effect sizes between EP protocols cannot be fully assessed.

Post-thaw embryo quality emerged as one of the strongest predictors of ART success. Higher proportions of TQ embryos, as well as A-grade ICM and TE, were observed in both positive pregnancy and LB groups. These findings align with previous studies demonstrating higher LB rates with superior post-thaw embryo morphology [17, 22, 23, 27, 29]. While some reports suggest that embryo morphology may not predict miscarriage or LB in euploid transfers [1, 5, 13, 18, 28], recent meta-analyses indicate that lower-quality ICM and TE grades are associated with reduced LB rates [23]. In the present study, subgroup analyses limited to mNC-prepared cycles confirmed the prognostic value of ICM grade, supporting the hypothesis that embryonic competence remains a key determinant of outcome even within euploid cohorts.

Re-expansion after thawing was more frequently observed in βhCG-positive cases but did not independently predict LB in multivariate analysis. Although several studies have associated re-expansion dynamics with improved implantation and pregnancy outcomes [10, 13, 15, 21, 39, 40], others have reported no association with LB [11, 14, 33]. Recent studies, including Bergin et al. (2024), have demonstrated that post-thaw changes in embryo morphology and re-expansion dynamics are significantly associated with live birth outcomes in single euploid embryo transfer cycles, supporting the importance of post-thaw embryological competence beyond chromosomal status [41]. These discrepancies may reflect differences in assessment timing, embryo quality, and study populations. Re-expansion reflects metabolic activity and TE function, which are essential for implantation; however, its absence in our study appeared to primarily predict negative pregnancy rather than LB, suggesting that surviving embryos may still retain implantation potential despite delayed re-expansion.

Day of biopsy, vitrification, and transfer also influenced outcomes. Day 5 embryos were more frequent among βhCG-positive cases, and Day 6 transfer was inversely associated with implantation. While many studies report superior outcomes with Day 5 embryos [17, 20, 27, 28, 30, 42, 43], others suggest comparable outcomes in euploid cohorts [5, 15, 18, 33]. Differences in embryo quality and synchronization with the endometrium may explain these inconsistencies.

Cryo-survival rates below 90% were uncommon in our cohort due to optimized vitrification protocols. Reduced cryo-survival was associated with poor embryo quality, necrotic areas, and excluded/extruded blastomeres, supporting previous observations that cellular integrity is critical for post-thaw viability and ART success. EB, reflecting embryonic self-correction mechanisms, has been associated with lower blastulation rates and poorer embryo quality. [24, 25, 44, 45], findings that were corroborated in the present study.

The major strength of this study lies in the consistent and meticulous execution of embryo culture and other IVF protocols over a prolonged period at a single center. This uniform approach, coupled with the involvement of two highly skilled embryologists in all IVF procedures, including TB, ensured methodological consistency and minimized inter-laboratory variability. Although embryo morphology assessment is inherently subjective, the use of standardized criteria and a consistent embryology team likely reduced variability. However, formal assessment of inter- and intra-observer variability was not performed, which should be considered when interpreting the findings. In addition, the discretion exercised by embryologists in selecting among available euploid blastocysts could have influenced outcomes. The decision to transfer a top-quality euploid blastocyst when multiple embryos were available may differ from cases where only a single euploid embryo of moderate morphological quality was present, potentially affecting implantation potential and the interpretation of morphokinetic predictors within euploid cohorts.

It should be noted that the mechanisms leading to BPL and CPL may differ, and the combined analysis used here reflects an overall rate of early pregnancy failure rather than distinct biological processes. One limitation of the present study is that BPL and CPL were analyzed together under the general category of PL. These two entities have different underlying mechanisms—with BPL often reflecting early implantation failure and CPL involving later gestational processes—and future studies should aim to evaluate them separately to better delineate their specific risk factors. Another limitation is that embryo selection among euploid blastocysts was based on embryologists’ discretion. Furthermore, a sensitivity analysis restricted to cycles with a single available euploid embryo could not be performed due to the retrospective nature of the dataset. Therefore, the potential impact of selection bias in cases with multiple euploid embryos cannot be fully excluded. Additionally, the indication for PGT-A was not included in the regression models, which may represent a source of residual confounding and should be considered when interpreting the findings.

The generalizability of these findings should be interpreted with caution. This study was conducted in a high-volume single center with standardized laboratory protocols and experienced embryologists, which may limit the applicability of the results to other clinical settings. Differences in embryo culture conditions, vitrification and warming protocols, embryo assessment practices, and patient selection criteria across centers may influence ART outcomes. Therefore, the observed associations may not be directly generalizable to all populations. Nevertheless, these findings may provide clinically relevant insights for centers using similar PGT-A and frozen embryo transfer protocols.

From a clinical perspective, these findings suggest that post-thaw reassessment of embryo morphology may provide actionable information even after euploid embryo selection. Parameters such as cryo-survival rate, ICM quality, and the presence of necrotic areas or excluded/extruded blastomeres may assist embryologists in prioritizing embryos for transfer when more than one euploid blastocyst is available. In addition, early post-thaw re-expansion dynamics may serve as a supportive indicator of implantation potential. Integrating these post-thaw embryological parameters with patient-specific factors, such as maternal age and endometrial preparation protocol, may improve clinical decision-making and optimize live birth outcomes in SEET cycles. These findings may also be particularly relevant in cases with multiple euploid embryos, where post-thaw morphology may guide the final embryo selection strategy.

## Conclusion

Morphological assessment before freezing alone may be insufficient to predict ART success in euploid embryo transfers. Comprehensive evaluation of post-thaw embryological parameters is essential for optimizing outcomes. In this large single-center cohort, embryo quality after thawing, transfer day, and cryo-survival characteristics influenced implantation, while younger maternal age (<35 years), higher post-thaw embryo quality, and the absence of necrotic areas or excluded blastomeres were associated with higher live birth rates. These findings highlight the continued relevance of embryological competence and maternal factors in achieving successful outcomes, even in the context of chromosomally normal embryos.

## Data Availability

The datasets generated and/or analyzed during the current study are not publicly available due to institutional and patient confidentiality restrictions but are available from the corresponding author upon reasonable request.
